# A qualitative programme evaluation of New Era, a mentorship programme for women leaders in sport

**DOI:** 10.3389/fspor.2026.1813783

**Published:** 2026-06-17

**Authors:** Kathryn Fortnum, Maria Carmen Almarcha, Zoe Harrison, Terrance Fitzsimmons, Véronique Richard, Samantha Mulcahy, Sharon Hinton, John Cairney

**Affiliations:** 1 The Health and Wellbeing Centre for Research Innovation, Human Movement and Nutrition Sciences, The University of Queensland, Brisbane, QLD, Australia; 2 Queensland Centre for Olympic and Paralympic Studies, The University of Queensland, Brisbane, QLD, Australia; 3 School of Business, The University of Queensland, Brisbane, QLD, Australia; 4 Office of 2032 Games Engagement, Office of the Vice Chancellor, The University of Queensland, Brisbane, QLD, Australia

**Keywords:** development, diversity, gender equity, inclusion, leadership, mentoring, sponsorship, sport

## Abstract

**Introduction:**

Despite the increase in women's participation in sport, women remain underrepresented in leadership roles across the global sport landscape. In response, governments and sport organisations have developed Women's Leadership Programs (WLPs) to foster women's development in sports leadership. Although these initiatives aim to strengthen women's confidence and leadership capabilities, existing evaluations often focus on programme structure rather than the relational processes through which women navigate, adapt to, and challenge gendered sport systems. This study examines one such WLP: New Era (SportsPro), a 12-month global programme integrating mentoring, leadership development, visibility opportunities, and community-building for women and non-binary sport executives and emerging leaders.

**Methods:**

A qualitative research design was employed using semi-structured interviews with 21 participants, including 13 programme alumni and eight Steering Group members. Data were analysed using thematic content analysis, combining manual and AI-assisted coding.

**Results:**

Findings were organised across (1) programme development and engagement, which captured the evolution of New Era, participants’ motivations for engaging, and the logistical and organisational conditions shaping participation. And (2) processes and perceived impacts of New Era, which captured the relational, personal, and industry-level experiences associated with participation. Across these points, participants described New Era as a relationally rich programme that supported visibility, expanded networks, personal brand development, leadership identity, and career progression. However, geographical barriers, uneven engagement, and ambiguity around programme goals constrained participation and raised questions about how to evaluate longer-term impact.

**Discussion:**

New Era illustrates the potential of relational, identity-based, and visibility-oriented leadership development to support women's leadership journeys in sport. Its contribution lies not only in individual development but in creating conditions for connection, recognition, and collective influence. However, broader organisational and sector-level impacts were harder to evidence or attribute directly to the programme. Future evaluations should adopt longitudinal and multi-level designs to assess whether perceived benefits translate into sustained career progression and organisational change across sport leadership.

## 1 Introduction

Women's participation in sports has grown substantially over the past three decades. Yet their progression into leadership positions (senior executives, board members, coaches, and technical officials) remains disproportionately low across all levels of the sport ecosystem (1–4). For instance, only 28.8% of executive board seats across International Olympic Federations are held by women, and just 11.7% of National Olympic Committees have a female president (5). Examination of this underrepresentation is not new: systematic reviews have identified systemic and interconnected barriers that prevent women from advancing into leadership roles in sport (6). These include entrenched organisational cultures that often favour male leadership, exclusion from informal yet influential networks, and pervasive stereotypes about who is “fit to lead.” Such obstacles are further compounded by the penalties associated with motherhood and caregiving responsibilities, which can limit women's career opportunities and slow their progression into senior roles (1, 7–9).

In response to these challenges, governments, sport federations, and related organisations have invested in Women's Leadership Programs (WLPs) as a strategy to enhance gender equity in sport leadership. Through developmental spaces such as workshops, seminars, networking events, and mentoring, these programmes aim to strengthen women's confidence, broaden their networks, and cultivate a stronger sense of identity and leadership agency as they progress towards leadership and decision-making roles (10, 11). Several global initiatives, such as the Women Leaders in Sports Institute (Australian Sports Commission), the Commonwealth Sport Women's Leadership Programme, the Women's Leadership Development Programme (Women & Leadership Australia), the Women Lead Sports Programme (The Association of Summer Olympic International Federations), and Female Leaders of Tomorrow (The Association for International Sport for All) illustrate the growing momentum behind these efforts (12). Across this expanding landscape, WLPs draw, implicitly or explicitly, on leadership approaches commonly associated with women's leadership practices, particularly Participatory (13), Distributed (14) and Transformational (15) leadership models that emphasise collaboration, relational practice, and the empowerment of others (16, 17).

However, this apparent shared orientation obscures important differences in the scope, depth, and underlying philosophical orientation of WLP. For instance, some programmes embody a “fix-the-woman” approach, focusing on the individual level, equipping women with the skills and confidence to succeed within existing systems. In contrast, others embrace a “fix-the-system” approach aimed at transforming organisational cultures, policies and structures that constrain women's advancement (6, 18, 19). Moreover, support has been delivered through structured mentoring arrangements, in which more experienced leaders guide participants’ development within a defined programme framework. As a result, much of the WLPs remains descriptive, focusing more on programme architecture than on relational learning processes through which women adapt to, navigate, and challenge entrenched systems (11, 20).

The New Era Programme, launched in 2022 by SportsPro, opens a space through which these gaps can be explored. Across a 12-month journey, 12–14 women and non-binary sports executives are connected and paired with a senior women leader from a diverse Steering Group. Rather than operating as a tightly structured leadership development programme, New Era offers a more relational and integrated model of women's leadership development that creates space for peer connection, experiences that enable visibility, and mentoring across the sports industry. Here, mentoring is relational, evolving through conversations between mentor and mentee in response to the mentee's context, experiences, and needs.

As mentoring sits at the heart of many WLPs, where participants are often supported through structured relationships with more experienced leaders, it is important to clarify what kind of support these programmes are designed to offer (21). Mentoring, sponsorship, and leadership development are concepts often used interchangeably in the leadership literature, but, in fact, refer to different forms of support. Mentoring is best understood as a dyadic relationship in which a more experienced person (the mentor) provides guidance, psychosocial support, career advice, and access to networks that the mentee may not otherwise reach (22). The purpose of mentoring is to strengthen an individual's confidence and skills and expand opportunities. Sponsorship is more explicitly opportunity-oriented, using the influence and networks to increase another person's visibility and open doors to advancement (23). Leadership is a social and organisational process: it involves mobilising people toward a shared direction, making decisions under uncertainty, navigating power dynamics, and influencing collective outcomes within complex systems (24, 25). Mentoring operates primarily at the interpersonal level, while sponsorship extends beyond interpersonal relationships to secure visibility and advancement within organisations. Leadership, in contrast, unfolds across multiple levels—individual, relational, organisational, and system-wide—and requires capabilities such as sensemaking, visioning, political negotiation, and the ability to work with paradoxes (e.g., promoting uniformity while valuing individualism) and competing demands (26, 27). Mentoring can support the development of these capabilities, but it does not constitute leadership. A mentee may become more confident or better connected, yet still lack opportunities to enact leadership, challenge organisational structures, or influence decision-making within an organisation. Distinguishing mentoring (a developmental relationship) from sponsorship (active advocacy for visibility and access) and leadership (a multifaceted practice embedded in context) enables a more precise understanding of what WLPs currently provide and where the limitations are.

Many WLPs focus primarily on mentoring and access to professional peer networks, and while important, they are insufficient to generate long-term impact. For women's leadership development to extend beyond individual support, programmes must also offer opportunities for women to engage in organisational- and system-level decision-making to achieve long-term impact (28). For example, interactions with senior leaders offer access to strategic conversations that shape the sector and strengthen women's visibility, influence and capacity to act beyond their actual role. Reverse-mentoring research suggests that such exchanges deepen leaders’ understanding of women's realities and constraints, thereby cultivating more informed and responsive leadership (29, 30). Through this bidirectional process, women expand their capacity to influence system-level dynamics, while leaders become *response-able*—to remain attuned to the reality of the other—in ways that can support structural change (31, 32).

It is here that New Era becomes particularly important. What distinguishes New Era from other leadership development programmes is the creation of opportunities for emerging women leaders to engage in conversation with senior women leaders across the international sport sector, fostering intergenerational exchange, visibility, and access to strategic knowledge. Specifically, the relational and reciprocal approach to mentoring, through which mentor and mentee evolve together, learning from each other and refining their leadership along the way. To better understand the impact of such a relational and reciprocal approach, the current study aims to explore both mentors’ and mentees’ perspectives on New Era; examine its perceived efficacy and developmental impact within the broader landscape of women's leadership in sport; and identify aspects of the programme that could be improved.

## 2 Materials and methods

To obtain depth of participant insights, a qualitative methodology was considered the most effective approach. Such an approach facilitates opportunities for participants to provide rich details of their lived experiences. Perspectives were obtained from current cohort members, New Era alumni (with current cohort and alumni herein grouped under the title ‘alumni’), and current members of the New Era Steering Group.

As outlined in the introduction, this study adopts a relational ontology on leadership development, grounded in the view that system properties are continuously interacting and evolving. Epistemologically, we adopt a constructionist stance, whereby knowledge is co-constructed through interactions between the researcher and participants (33). Guided by these assumptions, the study is situated within a pragmatic paradigm that foregrounds lived experience, an approach increasingly adopted in social science and business research (34, 35). A pragmatic stance enables a focus on what works, for whom, and under what conditions, within programs, in alignment with pre-specified research questions. Analysis was conducted in alignment with the principles of pragmatism Kelly and Cordeiro (35), specifically, an emphasis on actionable knowledge (ensuring the research has practical relevance); recognition of the interconnectedness of experiencing, knowing and acting; and considering enquiry as an experiential process, allowing for the mapping of experiences, consequences and meanings of social action.

A qualitative enquiry was conducted by a team of researchers at the University of Queensland (UQ). The team had no previous direct involvement in New Era; were not paid by SportsPro to conduct this work; have no conflicts of interest in relation to the programme (i.e., do not sit on any SportsPro boards); and were guided and supported by the staff responsible for designing and supporting the delivery of New Era, though no restrictions or interference were placed on the team during the conduct of this research. Ethical approval was obtained from the UQ Human Research Ethics Committee (approval number 2024/HE002422).

### 2.1 Data collection

All women who participated in the program, either as participants or steering group members between 2023 and 2025, received an email inviting them to take part in an interview about their experience of the program. The email included a link through which interested participants could provide consent and schedule an interview at a time convenient to them.

Interviews were conducted one-on-one using Microsoft Teams at a time determined by each participant. A semi-structured interview guide was utilised, informed by Kirkpatrick's Four-Level Model-Based Interview Schedule (36) and adapted from (10). Questions focused on reactions or impressions of New Era; learning (i.e., knowledge and skills acquired and the impact of these, both actual and perceived); improvement and application (i.e., application, or perceived application, of knowledge and skills gained in New Era to their personal ambitions or career goals); and results (i.e., participants perceived impacts of New Era on sporting organisations or the broader sports industry, with a focus on gender-equity). Alumni were also asked to share a story about a significant life event in their leadership journey, with an emphasis on one relevant to the New Era Programme. Participants’ stories were analysed only with respect to their interactions with and evaluations of the New Era programme. The presentation of story components outside the focus on evaluating New Era is beyond the scope of this manuscript. Interview guides are available in the Supplementary Material.

### 2.2 Data analysis

Interviews were recorded and transcribed verbatim. Content analysis using a thematic approach was applied to the data (37, 38). A thematic approach was adopted to enable a combination of latent and manifest coding, ultimately facilitating an understanding of women's experiences of the New Era Programme. The unit of analysis was sentences or paragraphs. The analysis process commenced with the first and second authors familiarising themselves with the data by listening to, reading, and re-reading all transcripts. Memos were kept independently by each author throughout this familiarisation process (38). Next, initial codes were generated independently via open coding and condensed into plausible, higher-order themes (37, 38). The second author adopted a traditional, manual approach to analysis on 50% of available transcripts (*n* = 7 alumni and *n* = 5 Steering Group). The first author concurrently generated initial codes from all transcripts with the support of AI, specifically ChatGPT o4-mini-high (39). AI has been shown to produce reliable results when instructed to adopt an inductive approach to content analysis and to generate in-depth themes that align with existing theoretical frameworks (40). Its use as an independent coder has therefore been endorsed when supported by humans with domain-specific knowledge who can appraise, critique, and adjust—via prompting—the output accordingly (40). The concurrent, independent approach to code and theme generation utilising AI was guided by Perkins et al. (41).

Analysis using ChatGPT o4-mini-high was conducted in July 2025. A project-specific GPT was configured for analysing data. To protect participant anonymity, only de-identified interview transcripts were uploaded (with names and other identifying details removed). The GPT was instructed to perform a thematic content analysis at the paragraph level. Prior to the analysis, the first author introduced the GPT to New Era by providing a link to the New Era programme website and prompting it to develop contextual familiarity with the programme.

Each interview transcript was then uploaded separately, and the GPT was asked to generate open codes with descriptions. These AI-generated codes were treated as provisional analytic suggestions rather than final codes. For each transcript, the first author manually reviewed the codes to ensure that they remained grounded in participants’ accounts and consistent with the research team's interpretive reading of the data. Then, the GPT was instructed to merge related codes into preliminary themes and generate theme descriptions. After reviewing the output, merged codes were added back into the GPT, which was instructed to condense them into higher-order themes. Following independent coding and preliminary theme generation, the first and second authors came together to discuss their findings as critical friends (42); a technique chosen to ensure reflexivity, transparency and interpretive rigour. Although both analyses captured the same key concepts, differences emerged in the level of interpretation, with some themes being more descriptive and others more interpretive. The authors adopted a socio-cultural constructivist perspective when considering and modifying the outputs generated by the GPT.

Where differences occurred, the authors returned to the transcripts to assess whether interpretations were grounded in the broader transcript context. The first, second, and fifth authors agreed to adopt a more descriptive thematic structure that remained close to participants’ experiences. The final codebook was then uploaded to GPT, which was instructed to apply it to all transcripts.

ChatGPT was therefore used as a supplementary analytic tool. Final decisions regarding theme development and interpretation remained with the research team. We recognise that AI-assisted qualitative analysis carries the risk of a lack of contextual sensitivity and interpretive bias. Those risks were mitigated through de-identification of transcripts, independent manual coding, critical-friend discussions, repeated returns to the data, and final human review of all AI-generated outputs.

## 3 Results

### 3.1 Participant demographics

A total of 21 participants took part in the study, including 13 alumni and 8 Steering Group members. Alumni ages ranged from 28 to 44 years (mean 34.3 years). Educational backgrounds varied, with six having Bachelor's degrees and the remaining seven having Master's degrees. Alumni were located across six countries, including the UK (*n* = 6), the USA (*n* = 2), Australia (*n* = 2), Ireland (*n* = 1), Canada (*n* = 1) and Germany (*n* = 1). Years of leadership experience in sport were <1 year (*n* = 1), 1–5 years (*n* = 7), 6–10 years (*n* = 4), and 11–15 years (*n* = 1). Current alumni roles within the sport industry spanned marketing/communications/fan engagement (*n* = 5), strategy/innovation/business operations (*n* = 3), legal/governance (*n* = 1), sustainability/purpose/social impact (*n* = 3), and events/operations (*n* = 1). Cohort representation was relatively balanced, with participants from the 2022-2023 (*n* = 4), 2023-2024 (*n* = 4) and 2024-2025 (*n* = 5) cohorts. Total alumni interview time collected was 490 min (average length 38 min).

The ages of the Steering Group members ranged from 43 to 55 years. Educational backgrounds were Bachelor's degrees (*n* = 4), Master's degrees (*n* = 3) and Honours degrees (*n* = 1). Participants were located in the UK (*n* = 5), the USA (*n* = 1), Spain (*n* = 1) and Singapore (*n* = 1). Steering Group members reported between 6 and more than 16 years of leadership experience, with the majority of members (*n* = 5) reporting over 16 years. Current roles within the sport industry fell across strategy/innovation/business operations (*n* = 4), marketing/communications/fan engagement (*n* = 3), and sustainability/purpose/social impact (*n* = 1). Steering Group members were involved in multiple cohorts (2022-2023, *n* = 5; 2023-2024, *n* = 5; 2024-2025, *n* = 8), with two involved for 1 year only, one for 2 years, and five for 3 years. A total of 290 min of interview data was collected from the Steering Group (average length, 36 min). Participants (alumni and Steering Group) have each been allocated a pseudonym to maintain anonymity.

### 3.2 Participant perspectives, experiences and perceived impacts

Themes are presented throughout the results section in a narrative analytic style that integrates participants’ perspectives across the dataset. This approach balances “showing” the data through selected verbatim extracts with “telling” the data through interpretation and synthesis (43), preserving participants’ voices while maintaining a coherent and engaging presentation of the findings (44). Quotes relating to recommendations are provided in Table 1.

**Table 1:** List of recommendations and supporting quotes.

Recommendations	Verbatism
Clearer articulation of goals, purpose, and beneficiaries	“Do we want to get more females into leadership or to get into senior leadership? Do we want to help females in sports to have more of a community feel? are they going to focus and try and have big impact in one area or have general impact across all areas?” – Mia, alumni
Clarification of expectations and structure of mentoring	“Some sort of a system that sets up regular intervals to catch up or a meeting schedule or something that can instill that discipline right from the start could be helpful.” – Ava, Steering Group
Support for mentors (training, wellbeing, clarity of role)	“It would be lovely to have a unified approach so that the mentees I have under my wings get the same as others. I like that expectation to be high, but the same for all of us.” – Rachel, Steering Group
Optimisation of mentor–mentee matching and relationship building	“Maybe it's understanding the mentors better to know our style… matching people with the right style. That could be a good thing to do.” – Alana, Steering Group
Enhancing and leveraging digital community (platform, jobs hub, resources)	“New Era could be an online platform or something that pools or collates resources on different topics something bite-size and digestible, quick bits of advice or reading material.” – Kelly, alumni
Ongoing involvement and exposure for alumni (opportunities beyond programme year)	“Hopefully they remember that they have a large pool of women now who are happy to and are confident to speak at these events… just more opportunities to stay involved.” – Kara, alumni
Supplementing mentoring with focused development opportunities	“Maybe… more targeted training some soft skills nobody gives you a guidebook on how to be a leader but I guess it depends on New Era's long-term goal.” – Sabrina, Steering Group
Strengthening connection and relationship building (cohort, alumni, Steering Group, whole community, global)	“Facilitating more engagement through the cohort themselves… that was probably almost the most enjoyable part of it. I didn't think about connecting with the cohort as much as I did, and that was very valuable.” – Lisa, alumni
Engaging organisations of current cohort	“it’d be nice for the companies to know the mentors” – Alana, Steering Group
Leverage New Era community and networks (alumni, Steering Group, collective action)	“the biggest thing is the alumni… how are we using that network and that power for good, and what are we trying to achieve… together, what would we want to campaign for? it's a collective action because that will be like the ‘think tank,’ the experts, the data, people coming to us, there's so much potential, there's no one else doing this. So, we’re completely leaders, but we’re probably just not utilising it in the right way.” – Alana, Steering Group
Leveraging partnerships and sponsorships	“If you ever brought in sponsors, it would be great if they could help cover [travel] cost because it's a lot to ask of small businesses to pay for leaders to attend events.” – Maria, alumni

#### 3.2.1 General impression of the programme

General programme impressions from both alumni and the Steering Group described their participation in New Era in terms of gratitude, pride, and a strong sense of privilege. For many alumni, New Era arrived at a pivotal moment in their careers, enhancing their confidence and providing visibility and a sense of community. Steering Group members echoed these reflections, expressing pride in contributing to an initiative they felt “changed people’s lives” and praising SportsPro's bravery in establishing a space dedicated to women in sport leadership. The programme was regarded as unique for mentoring practice in the sector, while the professionalism and support of the SportsPro team were recognised as central to successful delivery and impact.

#### 3.2.1 Programme development and engagement

**Figure 1:**
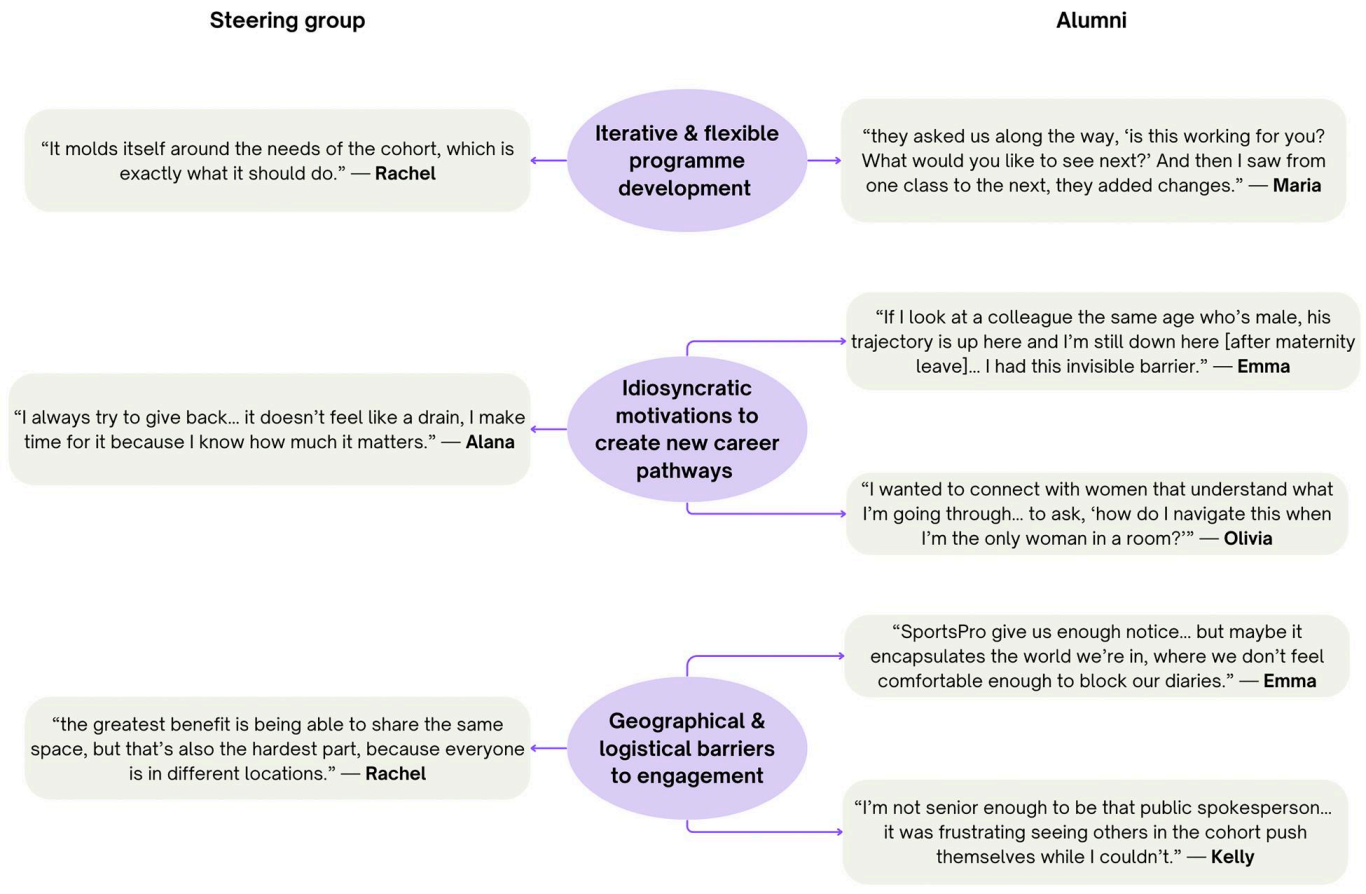
Participant quotes relating to programme development and engagement.

Programme development and engagement focus on the development and evolution of New Era, the factors motivating alumni and Steering Group members to engage, and the values and experiences shaping their involvement. It also examines how geographical location, logistical constraints, and organisational contexts influenced patterns of participation and engagement. This content will be covered through three sub-themes: Iterative and flexible programme development, idiosyncratic motivations to create new career pathways, and Geographical and logistical barriers to engagement (see Figure 1).

##### 3.2.1.1 Iterative and flexible programme development

Participants reflected on New Era as an evolving programme that has improved across successive cohorts, has been built in dialogue with its community, and is constantly adapting to changing needs. Alana (Steering Group) recalled the origins of the programme, when conversations about creating a community for women in sport were informal and uncertain, wondering what exactly they should do, then reflecting on how the programme has since grown. Sabrina, who had been on the Steering Group from the start, recalled early iterations as experimental, for example, having the opportunity to speak with all members of the cohort, relating it to “speed dating”. Jessica (Steering Group) similarly described the “revolving door” of the first year, where relationships felt too brief to build trust. Both noted how the change to assigning smaller groups of mentees to each mentor transformed the depth and quality of engagement.

Participants also highlighted the openness of the Sports Pro staff to receive feedback about New Era. For Maria (alumni), this responsiveness was derived from co-creation, whereby Sports Pro were continually seeking feedback and adapting the programme from one year to the next. She interpreted this openness as generosity. Rachel (Steering Group) described programme development as “a fluid and flexible approach… not rigid in any way”, highlighting how quickly ideas were turned into action. She suggested a session on balancing motherhood and professional careers, which was organised almost immediately, reflecting how this ensured New Era met the needs of each cohort.

##### 3.2.1.2 Idiosyncratic motivations to create new career pathways

Some alumni were encouraged to apply by colleagues or managers with the aim of facilitating professional development opportunities. For others, entry into the New Era appeared to result from moments of career transition or crisis, rather than from periods when things were going well, as reflected by Ava (Steering Group). After several years in a flat organisational structure, Emma (alumni) applied to create opportunities to take the next step in her career. She also reflected that family circumstances and career interruptions shaped her motivations, stating that she felt her career trajectory was much flatter than that of a male of the same age after returning from a three-year work hiatus. For her, New Era provided a way to reestablish a career trajectory that had felt disrupted by maternity leave. Mia and Kara (alumni) applied as an act of resistance against structural inequalities. Mia worked in sports analytics and described how male-dominated hiring panels repeatedly undermined her expertise and made her feel like she wasn’t welcome. For her, joining the programme became a way to “pave the way for others” and to channel frustration into leadership development. Kara similarly traced her motivation to gendered experiences of being undermined, having previously been told by a manager that she needed to behave differently as a leader so people would know she was in charge. Olivia (alumni), meanwhile, described joining to find solidarity with women facing similar isolation.

Members of the Steering Group joined New Era and remained engaged for several reasons. Rachel’s motivation was to expand her network and stay connected to emerging voices in the industry. She also felt energised from conversations with alumni. Heather, who identified as a woman of colour, had deeply personal motivations and reflected on her positive experiences with mentors who could ground and motivate her or serve as an example. Alana indicated that New Era aligned with her value of ‘giving back’ and felt that it was an important programme for women in sports leadership.

##### 3.2.1.3 Geographical and logistical barriers to engagement

With SportsPro (and therefore New Era) based in London, access to live events, the capacity to fully engage with the program, and cohort connection were easier for those living locally than for international New Era community members. Various financial and logistical barriers were described by alumni members. Maria (alumni) admitted she could not afford to travel from her base in a small startup, while Emma (alumni) reflected on pushing hard for the budget to attend SportsPro Live in London, but being refused. The resulting divide was described by Mia (alumni) as “the UK vs. all of the rest of us….” Connection challenges extended to the Steering Group as well, with Heather reflecting that in more than three years she had “never had the opportunity to actually go and meet” her mentees at formal gatherings, instead relying on lucky encounters or arranging *ad hoc* meetings when in London. Rachel (Steering Group) reflected that meeting face-to-face was beneficial for the programme, but challenging to achieve.

Alumni such as Abigail were also a bit frustrated by low cohort engagement in Connect sessions, which they interpreted as a symptom of broader workplace pressures. Conversely, Emma (alumni) reflected on time zone barriers to participating in such sessions, recalling attempting to join Connect sessions at 3:30 a.m. while balancing family commitments: “These are the things where sometimes the priorities have to shift.” Kelly (alumni) described being prevented from taking up opportunities available to her because of restrictive organisational cultures that, for example, stopped her from moderating a panel.

#### 3.2.2 Processes and perceived impacts of New Era

**Figure 2:**
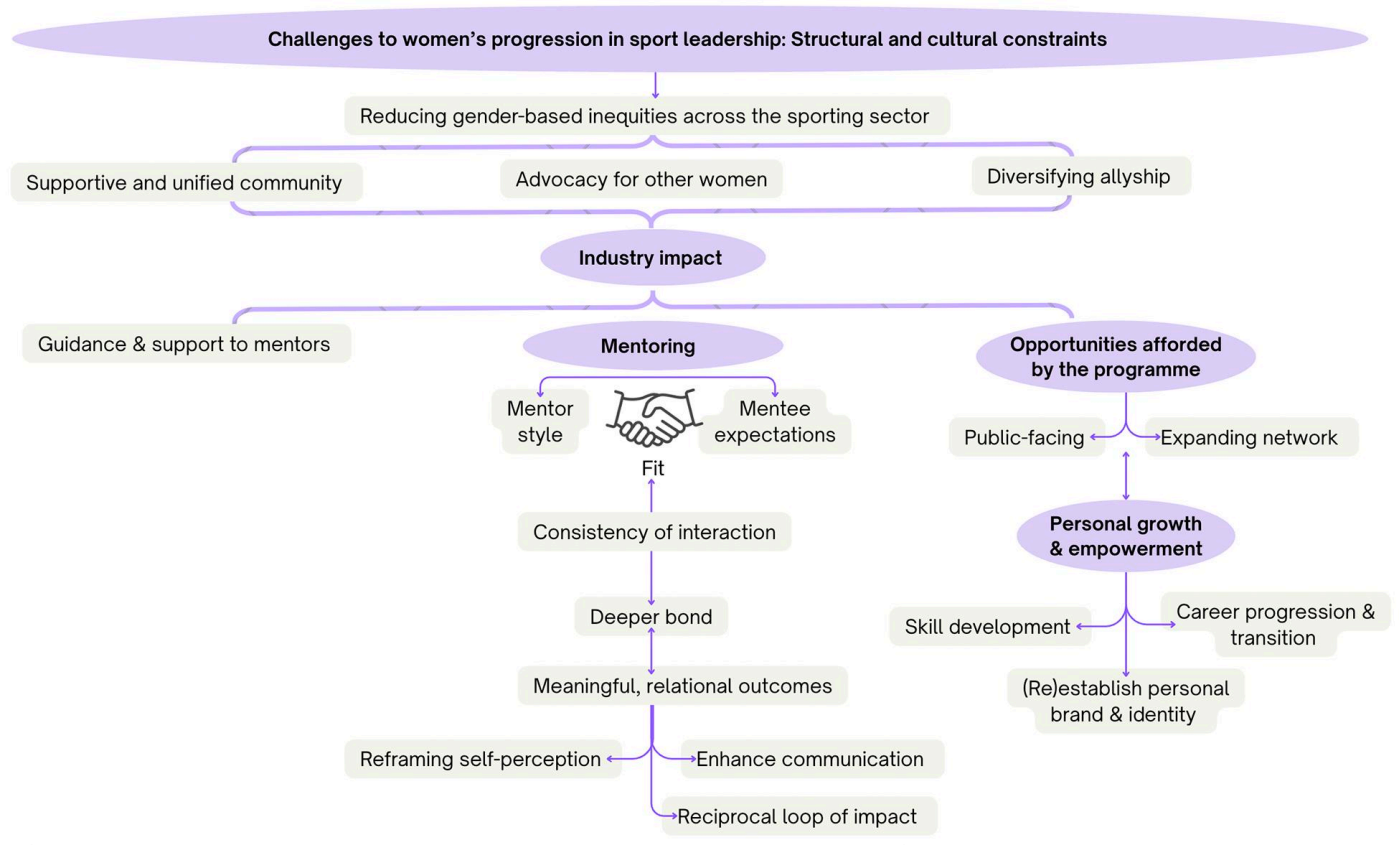
A holistic representation of the programme's processes and perceived impacts.

Once participants were engaged, and throughout the evolution of New Era, various processes unfolded that shaped their perceptions of its relational, personal, and industry impacts. Figure 2 illustrates the relationships between the units of meaning presented below and provides a more holistic representation of the programmer's processes and perceived impacts.

##### 3.2.2.1 Mentoring or mentor-mentee relationship

Mentoring was a central aspect of New Era, and throughout the programmed these experiences were shaped by varied mentoring styles, the level of organizational scaffolding provided to mentors, the mentor–mentee pairing process, expectations, and the fit between Steering Group approaches and alumni needs (See Figure 2).

###### 3.2.2.1.1 Freedom to express individual differences led to varied mentoring styles

Embracing an autonomy-supportive approach to mentoring, BLINDED] did not impose a specific mentoring style. Consequently, mentoring practices varied, reflecting differences in Steering Group members’ personalities and philosophies, as well as the evolving needs of alumni, as described by the following Steering Group members. Angela (Steering Group) took a broad approach, by frequently conducting skills mapping exercises that encouraged women to consider where they are now, where they want to be and what is required to get there. Rachel (Steering Group) also focused on providing practical support, particularly on career progression and connecting alumni with relevant people in the industry. Sabrina (Steering Group) explained that her style involved asking “the hard questions,” designed to provoke reflection and reframing of challenges. Jessica (Steering Group) took a similar approach to Sabrina and felt that what she offered was “diversity of thought and different perspectives”. For example, she advised one woman to seek the opportunity to be “seconded to one of her clients” when “she had nowhere to go in terms of progression”, resulting in a 12-month secondment that was appreciated. Alana indicated that she likes to focus on women's personal brands. Heather (Steering Group), by contrast, positioned herself as part of the mentees’ extended support networks.

###### 3.2.2.1.2 Freedom within structure: mentors also need guidance

While no specific mentoring style was imposed, Steering Group members reflected upon the scaffolding provided by New Era to support their mentoring practice. Rachel (Steering Group) described the “prompts, guidance, reminders” as enabling her to balance mentoring with professional demands. For others, however, support felt less systematic. Ava (Steering Group) noted that while “fantastic support” was available if she reached out, the process felt *ad hoc*. She suggested that more structure would have been helpful, especially in the early stages of the programme. Sherry's (Steering Group) account was more critical, noting she didn't receive any ongoing contact.

###### 3.2.2.1.3 A pairing process shaping mentor–mentee fit (or misfit)

The success of mentorship did not rely solely on mentors’ styles or the guidance they received. At the mentor–mentee relational level, success largely depended on the relational fit and quality of connection between alumni and their mentors; when mentors’ approaches aligned with mentees’ needs, the relationship was most impactful. Some alumni members felt energised by the Steering Group, which pushed them to think differently, while others sought reassurance and solidarity. Sherry (Steering Group) reflected on challenges arising from the mentor-mentee pairing process (conducted by New Era staff, with a focus on aligning alumni members with Steering Group members in the same sport area, or as requested by alumni). She detailed the process as “ occasionally forced”, which sometimes resulted in mismatches. Such mismatches, she explained, made it harder to establish the trust that effective mentoring relies upon. Alana (Steering Group) reflected that there may be a benefit in “understanding the mentors better to know our style… to know what our strengths and weaknesses are”, allowing cohort members to access Steering Group members based on the area of development being sought or the coaching style used. Sabrina (Steering Group) emphasised the need for careful mentor selection and, more broadly, evaluation, given the important role mentors play for mentees.

###### 3.2.2.1.4 Fit in relation to mentees’ expectations

The fit (or misfit) was further shaped by mentees’ expectations of the mentor–mentee relationship. For some, structure and explicit expectations were key; for others, it was the open door of informal, supportive contact. Some Steering Group members, like Heather, place the responsibility on mentees to initiate contact. Others adopted a more proactive stance, keeping in touch with their mentees through WhatsApp or informal check-ins alongside scheduled sessions. For some alumni, the flexibility in their meetings was liberating, allowing the relationship to unfold organically, but for others, it created uncertainty and frustration when expectations were not met.

###### 3.2.2.1.5 A deeper relational bond emerging from consistent connection

Once relational fit was established, continuity of connection was identified as critical to mentoring success. Sherry (Steering Group) recalled that consistency mattered most to the success of mentoring. Jessica (Steering Group) highlighted the long-term bonds she established with her mentees. Kelly (alumni) also reflected on the importance of connection in relation to mentor availability and perceptions of commitment to the process, highlighting that this variation was detrimental to her experience. Though few in number, alumni with experiences similar to Kelly's were slightly frustrated or disappointed and felt they may have missed out on the full benefit of the programmed.

##### 3.2.2.2 The mentor–mentee relationship as a driver of meaningful relational outcomes

Overall, the quality of mentoring provided by Steering Group members was highlighted as a defining strength of New Era. Alumni expressed awe at the opportunity to learn from leaders whose reputations were well-established in the sports industry, with a sense of high calibre extending beyond professional achievements to personal credibility. Mia (alumni), who had never experienced formal mentoring before, appreciated the “outside perspective” and felt that her relationship with her mentors was “life-changing.” For Olivia and Lisa (alumni), the resonance of shared experience was particularly powerful, instilling in them a confidence that their challenges were both valid and surmountable.

###### 3.2.2.2.1 Reframing self-perception during times of adversity

Alumni found New Era transformative during periods of low confidence, burnout, or disengagement with their careers, with mentoring and relationships with the Steering Group particularly credited for this. Abigail (alumni) reflected improvements in confidence from speaking with members of the Steering Group. Mia (alumni) described how mentoring reframed her self-perception, building her confidence and challenging internalised doubts.

###### 3.2.2.2.2 Enhance communication

Practical techniques learned through mentoring that built alumni confidence included day-to-day communication skills. Elizabeth (alumni) reflected on advances in her communication skills and strategies for approaching important conversations. Mia emphasised improvements in her ability to influence and persuade others, which she found empowering. Jessica (Steering Group) identified improvements in confidence and empowerment within the broader New Era community through opportunities for honest and transparent conversations that focus on people's past successes and failures, for example, during an ‘Ask me anything’ session at New Era Live.

###### 3.2.2.2.3 A reciprocal loop of impact

Steering Group members described New Era as a source of their own growth. Jessica summarised: “I’ve learnt as much from my cohort as they’ve probably learnt from me.” Heather (Steering Group) reflected that mentoring prompted her to reconsider her own leadership: “By speaking to mentees, you go back and reflect on how you’re managing your team.”

#### 3.2.3 Opportunities afforded by the New Era to raise the alumni profile

Beyond mentorship, New Era offered participants a range of opportunities. Engagement with these opportunities is first described, followed by the perceived impacts on participants.

##### 3.2.3.1 Public-facing opportunities to share knowledge and experiences

Panels, webinars, podcasts, and articles were described as vital opportunities for profile raising and demonstrating leadership. Mandy (alumni), when first asked to write an article, declined out of self-doubt: “I thought, ‘why would anyone want to listen to me?’” Only after the New Era staff encouraged her a second time did she agree. Looking back, she saw it as a defining moment to “find her voice”. Mia (alumni) capitalised on these opportunities, stressing the importance of being visibly represented on industry stages and in published content on future opportunities. The benefit of utilising New Era events to facilitate safe spaces for personal growth and exposure was also reflected on. The importance of appropriately preparing alumni members, however, was emphasized. Angela (Steering Group) described being well-prepared through briefings, which supported her confidence, along with the confidence of the alumni she was presenting to. However, Alana (Steering Group) expressed concern that alumni members placed on conference stages were sometimes underprepared. She worried that without proper preparation, opportunities intended to empower could backfire by undermining confidence.

##### 3.2.3.2 Possibilities to expand the network internationally

New Era was also recognised for opening doors to new contacts, industries, and global markets, often providing introductions that would not otherwise have been accessible. Claire (alumni) credited the advocacy efforts of Steering Group members for this. Mandy (alumni) felt more well-known in the industry, both on online platforms (e.g., LinkedIn) and at events. Kara (alumni) reflected that the benefits were evident to the cohort through increased awareness of their expertise. Alana (Steering Group) indicated that New Era allowed alumni to celebrate and promote themselves on social media platforms such as LinkedIn, which was associated with increased credibility and recognition of their expertise. Benefits of exposure-based opportunities were also felt by members of the Steering Group, reflecting that it broadened their understanding of diverse sports structures, markets, and cultures. Ava, a member of the Steering Group, felt this was helpful because “sport is truly a global language”.

In some cases, involvement in New Era raised participant organisations’ visibility, opening business opportunities and expanding their international networks. Lisa (alumni) reflected that Steering Group contacts provided *“*a chance to show our product internationally”, indicating how “helpful” this has been. The organisation Mandy (alumni) worked for valued the increased “second-hand exposure” that stemmed from having their name associated with New Era and the increased visibility that came from her presentation at international conferences. Olivia (alumni) reflected on the increased international awareness of her work and the potential benefits for increasing the number of women in the sporting industry globally and expanding global sporting opportunities.

##### 3.2.3.3 Personal growth and empowerment

Through engagement with these opportunities, participants reflected on their personal growth and developing sense of empowerment.

###### 3.2.3.3.1 Skill development emerging through engagement with opportunities

Mandy (alumni) felt empowered to access the network available to her through developing the skills and confidence to ‘network’. Some alumni felt they developed ’soft skills’. For example, Lisa (alumni) showed a newfound empathy for supporting parents, as this is not something she has had firsthand experience with. For Mia (alumni), New Era helped her to recognise that she wasn't empowering her team as much as she could be or wanted to as she progressed into more senior roles. Mandy (alumni) felt that New Era helped her “transition from a doer to a leader”. Kelly (alumni) saw New Era as a catalyst for focusing on, developing, and translating specific skills into practice.

###### 3.2.3.3.2 (Re)establishment of personal brand and identity

New Era was also seen as a tool for recognising and shaping (or re-establishing) women’s personal brands, distinct from their organisational identity. Mia (alumni) articulated this, “Going through the programme, it helped me build my brand again. I think I had lost it, I had intertwined it with my current job and the organisation. It just reinvigorated that, ‘this is what I want to do, this is who I want to be, I want to help others”. Additionally, perceptions of ‘what leadership looks like in sport’ shifted, and alumni were enabled to develop a stronger sense of themselves as leaders by learning to embrace their own style and push back against gender-based leadership norms. Kara (alumni) reflected “getting that reassurance that I didn't have to change who I am to become more senior confidence that leadership styles that I gravitate towards are just as important and powerful as those typically more assertive and dominating leadership roles that you typically see in sport. I realised that I can bring in elements of that and dial that up when I need to, but there’s also power in a more sensitive approach and nurturing talent.”

###### 3.2.3.3.3 Career progression and transition

Both alumni and the Steering Group credited New Era with helping alumni secure new roles, promotions, or career-defining projects. Steering Group members were often used as sounding boards during periods of career-related change or were leaned on to “see the wood from the trees” and to “get some clarity over the decision that was in front of me”, as Kelly (alumni) reflected. Steering Group members were also seen as relatable role models for new and working mothers, with alumni finding reassurance in their experiences and examples of balancing a demanding career with family life, including navigating sensitive organisational contexts relating to parenthood, preparing for leave, and re-entering the workplace with confidence. Connections with other cohort members experiencing similar parenting transitions were also a pertinent source of support and inspiration for alumni*.*

#### 3.2.4 Industry impact

In addition to relational and personal outcomes, women also reflected on the broader industry impact generated by New Era. This theme captures participants’ perceptions of New Era's broader influence on the sporting sector, including community building, advocacy, and ally ship.

##### 3.2.4.1 Building a supportive and unified community of women in sport

A strong sense of community and solidarity, fostered within a male-dominated industry, emerged as a key contributor to broader industry impacts. Kara (alumni) reflected on the sense of belonging she felt from seeing other women walk the walk, “seeing women on stage… it just makes you feel like you belong”. Maria (alumni) reflected on how “refreshing” it was to be in a room full of other “minority” people (i.e., women in sports leadership) who share a shared, unspoken understanding. Amanda (alumni) reflected on the sense of trust established within the peer network based on shared values and experiences. Kara (alumni) articulated the sense of safety the peer cohort provided, especially when attending events. Similarly, Alana (Steering Group) reflected on the importance of providing spaces where women can share the gender-related challenges they face in the industry.

##### 3.2.4.2 Advocacy for supporting other women in sport

A strong sense of advocacy and the importance of being a diversity champion were instilled in alumni, with many reflecting that they had the desire to “pay it forward” and support up-and-coming women in the industry through mentoring and passing on their lessons. Olivia (alumni) emphasized the importance of advocacy in a male-dominated industry to help women break in and progress up the leadership pipeline. Elizabeth (alumni) reflected that it was a “unique honour” to be in the programmed. She recognized that many women in her organization would not have the opportunity to access it, so she is currently working on a way to bring in “a team of experts from outside” to support aspiring women leaders. Mia (alumni) did not want other women to experience the challenges that she went through, which fueled her passion to advocate for change and support other women. Jessica, a member of the Steering Group, indicated that “it's that army of people” being created through New Era that can carry the sense of “purpose around driving change within their own organizations”, meaning that it is “less about one individual trying to champion change”.

##### 3.2.4.3 Beyond women: diversifying ally ship

Alumni members contemplated the importance of ally ship on gender-based change. Claire liked that “more and more people are getting involved and not only women, but male allies”, as she felt that “support from our male counterparts” was important in changing the landscape for women in sports leadership. Amanda reflected that the integration of males into workshops was a positive, enabling, and facilitating ally ship, but emphasized the importance (and the difference) of New Era being “completely women-led”.

##### 3.2.4.4 Reducing gender-based inequities across the sporting sector

Together, the benefits of community building, advocacy, and ally ship were emphasized by alumni members as a potential avenue for reducing gender-based inequities across the sporting sector. Abigail (alumni) felt that New Era gave her “hope”, “something to aspire to”. Claire (alumni) realized the “potential that there is for women in leadership in sport”. Becoming a “visible” and “recognizable” brand in the sporting industry through New Era events was identified by Rachel (Steering Group) as having potential to help “women to consider [a] career in sports or to keep them in it”. Ava (Steering Group) indicated that the “empowerment” impact of New Era on participating women, through seeing and hearing the experiences of more senior women in the industry, would have flow-on effects to “change those equity numbers” in leadership in sport. increased visibility for women, and the perceived “permission” to “see-it-be-it”.

#### 3.2.5 Challenges to women's progression in sport leadership: structural and cultural constraints

Members of the New Era community flagged how structural and cultural constraints continue to shape women's progression in the sports industry. These barriers were not always presented as explicit acts of exclusion. Rather, through the persistent assumptions about women's authority and legitimacy. Rachel (Steering Group) pointed out that some women may not “lean into careers in the sports industry” for “preconceived ideas.” According to Kara (alumni), such ideas “are ingrained in us,” for instance, to assume that men are in charge when they are present in the room alongside women. Angela (Steering Group) even described a “lack of respect… about what we’re doing and why we’re doing it and the value we bring to the industry” when representing New Era on stage at New Era Live events. Amanda (Steering Group) described a continuing “ceiling for women CEOs in sport,” which limited the visibility of women in the most influential positions and, in turn, narrowed what women leaders could imagine as possible. In part because some aspects of women's development in sport “remain pretty tough to crack”—Jessica (Steering Group).

Participants also highlighted how exclusion can be difficult to name because it occurs through subtle and intersecting forms of disadvantage. Amanda (alumni), a non-native English speaker, described how “the work environment can be hostile” because “people find ways to exclude you or to shut you down in different ways.” For her, this exclusion was not easy to evidence: “it's in the details, it's hard for you to point this out and shout about it.” She reflected on not knowing which aspect of her identity was being judged: “Is this because I’m a woman? Is this because I’m from the Global South, or I’m not a native [English] speaker? I am a white person, but if I were a Black person, would that be different or add another layer? It might be because of the combination, but we will never know.” Her account illustrates how gender inequality may be compounded by language, nationality, race, and other social positions, making exclusion harder to challenge.

Alana (Steering Group) provided an example of gendered assumptions during an interview for a senior role. She was asked whether being menopausal would affect her capacity to perform as chief executive, which she responded by reminding the interviewer, “that's illegal to ask me.” As a result, she was perceived as “too outspoken.” Together, these examples show how women's legitimacy in sport leadership can be undermined through subtle gendered and cultural assumptions.

#### 3.2.5 Recommendations

Recommendations to strengthen future programmed delivery were provided by members of the New Era community and are summarized in Table 1.

## 4 Discussion

Women's representation in leadership positions in sport remains low, despite global policy commitments to gender equity (40). In response, WLPs have been developed to expand women's access to leadership opportunities and to develop their leadership capabilities. This study examined one such initiative, New Era, a global year-long programmed delivered by SportsPro that combines one-to-one mentoring, leadership development, networking, and opportunities for industry visibility for women and non-binary sports executives and emerging leaders. The purpose of this research was to explore the perceived efficacy of New Era, assess its perceived impacts on the broader sporting landscape in relation to gender equity, and identify recommendations for programmed improvement from the perspectives of alumni and the members of the Steering Group.

### 4.1 Perceived value of New Era within the women's leadership programme landscape

Across the participants, New Era was widely regarded as a prestigious, adaptive programmed that evolved with each cohort. The global nature of New Era was broadly recognized as a strength, though participants from outside London reported challenges with connection, engagement, and accessing opportunities. Mentoring, provided by a high-calibre Steering Group, was a standout, with the quality of relationships and the logistics of planning and engaging with sessions influencing success. Alumni described development across several domains, including confidence, visibility, expanded networks, authentic leadership, personal brand development, and career progression, aligning with existing evidence that WLPs enhance women's leadership self-efficacy, identity alignment, and visibility (11, 19, 45, 46). While participants described clear perceived benefits for their own development and professional networks, broader organizational and industry-level gender-equity impacts were harder to observe or attribute directly to the programmed.

### 4.2 From mentoring to sponsorship: opportunities and leader identity

Women engaged with New Era seeking mentorship in response to diminished confidence within the sporting industry, frequently associated with gendered experiences of being undermined; career disruptions and interruptions, commonly associated with parenting responsibilities; and a desire to change the landscape for other women in sports leadership. However, findings suggest that New Era extended beyond traditional conceptions of mentoring in WLPs, with many alumni describing support that resembled sponsorship.

One difficulty in analysing the experiences of participants in an autonomy-supportive approach with few defined boundaries is that mentors engaged in a range of behaviors spanning multiple paradigms in the literature. These distinctions, and their overlaps, remain contested in the literature (47). Interactions between mentors and mentees drew upon overlapping features of mentoring (48, 49), coaching (50), and sponsorship (47). While doubtless the programmed offered mentoring experiences, such as holistic personal and professional development (48), the short-term nature of the engagement also shared features of coaching, which is characterized by a shorter duration, goal focus and concentration on addressing one or two key areas for development. Likewise, the extended interaction between a smaller subset of mentors and mentees, particularly when the mentor could advocate on behalf of the mentee and leverage their connections, could more accurately be described as sponsorship (47).

One-on-one sessions with the Steering Group were described as critical to meeting alumni’s goals. While framed as ‘mentoring’, which traditionally responds to the developmental question *“How can I support your development?”*, members of the New Era community described interactions that extended beyond the boundaries of conventional mentoring. In these cases, the Steering Group acted more like ’sponsors’, using their networks, credibility, and organisational influence to open doors, increase visibility, and connect alumni with senior leaders (23). These actions speak to the more instrumental question *“How can I use my influence to move you forward?”* The blend of relational mentoring support and the opportunity-enhancing functions of sponsorship encouraged alumni to consider a broader systemic question: “*How can I support the success of a larger collective or organization?”*, a hallmark of contemporary leadership practice oriented toward guiding collective action (51).

Mentoring and sponsorship behaviors also appeared to facilitate identity development—the growth of a leader through recognising oneself and being recognized by others as a leader (52, 53)—which provides the social affirmation and visibility to strengthen leader identity. Identity development was reflected in participants’ language about “developing my personal brand” and “seeing myself as a leader.”

### 4.3 Developing authentic leadership: support, role models and intersectional networks

Women's leadership development occurs within gendered organizational contexts. Many participants described navigating an “authenticity paradox,” in which leading in alignment with their values collided with entrenched gendered norms of leadership (53, 54). New Era appeared to alleviate this tension by offering safe, women-only spaces that supported open reflection and honest discussion of challenges. Such environments have been shown to foster psychological safety and facilitate open discussion and the communication of experiences (17, 55, 56). Within women-only settings, alumni also encountered diverse leadership styles that broadened their understanding of what effective leadership looks like and supported the development of their own authentic leadership approach. New Era's emphasis on authentic leadership and self-reflection resonates with contemporary relational and values-based leadership perspectives (57, 58) and with research in Authentic Leadership Theory, which positions identity and value-driven behaviour as central to effective leadership development (59, 60).

As a programmed for women, led by women, New Era offered alumni the opportunity to identify inspirational role models in the industry. The impact of this on alumni can be understood through Role Congruity Theory and Social Network Theory. Role Congruity Theory highlights how leadership is often equated with masculine traits (“think leader, think male”) and how this association contributes to women's perceived “lack of fit” in senior sport leadership roles (61). By forming relationships with successful, authentic women leaders, alumni may have gained protection against the consequences for women leaders associated with role congruity theory, including gender-based stereotype threat, which is associated with reduced feelings of belonging, diminished confidence, and disidentification from the industry (62).

Beyond role modelling, the established relationships between members of the New Era community reflect the benefits identified in Social Network Theory. Access to social networks frequently determines opportunities for advancement within the sporting industry. The bridging of ties among alumni, senior women leaders, and industry platforms was described as opening doors for some alumni, partially mitigating the effects of male-dominated structures that continue to restrict women's access to senior positions in sport (63). However, whether these experiences led to wider structural change was not explored in this study.

The global design of New Era also created the conditions for intersectional learning. Bringing together women across nationalities, races, classes, and organizational cultures cultivated awareness of how intersecting identities and contextual factors shape leadership trajectories (64). Such exposure appeared to allow alumni to recognize and navigate leadership-related constraints across diverse settings (18, 65). This is broadly consistent with research in the general organizational psychology literature, which posits that leadership development is a *multi-level phenomenon* that cannot be understood solely at the individual level; instead, it should be studied across **i**ndividuals, dyads (relationships), and teams/organizations (66).

### 4.4 Participant-informed recommendations for women’s leadership programmes: individual, relational and organizational perspectives

Leadership development research suggests that high-quality programmed are distinguished by coherent design across levels of analysis: organizational pathways (e.g., clear goals and opportunity structures), relational processes (e.g., mentoring and networks), and individual learning (e.g., confidence and skills) (66). Accordingly, participants’ recommendations of New Era can be read as a call to strengthen programmed coherence across these levels and offer practical guidance for the design and delivery of future WLPs.

At the individual level, alumni advocated to supplement mentoring with targeted skill-building, such as communication, negotiation, and leadership skills, which remain underdeveloped in many WLPs despite their importance for senior-level readiness (56, 67).

Clarifying expectations around mentoring was a central recommendation at a relational level. Some members of the Steering Group anticipated regular meetings, while alumni were not sure how often they should initiate contact and often hesitated to “bother” their allocated Steering Group members. Both alumni and the Steering Group alike suggested that establishing scheduled touchpoints or a shared meeting structure from the outset could help set mutual expectations. This is consistent with research showing that mentoring quality improves when programs guide roles, responsibilities, and communication norms, and when mentors receive support to enact these roles effectively (22, 49, 68).

The need for structure may be especially relevant in online and distributed mentoring contexts, where ambiguity around expectations can exacerbate social and psychological barriers to engagement. Hughes et al. (69) show that when communication norms and interaction structures are unclear, mentees may experience hesitation, reduced trust, and lower perceptions of social presence—factors that discourage proactive contact and weaken collaborative relationships. Establishing agreed-upon schedules and interaction protocols can therefore help normalise contact, reduce perceived imposition, and foster psychological safety within mentoring relationships. Kang et al. (70) also highlight that effective online mentoring benefits from intentional facilitation and structured phases, particularly during the early stages of relationship formation. Their analysis indicates that mentors who actively scaffold interaction—through regular check-ins, goal clarification, and timely feedback—are more likely to sustain engagement and support mentee development over time. These findings reinforce the value of embedding structured mentoring processes within programmed design, rather than relying on informal or *ad hoc* interactions. Participants further advocated to supplement mentoring with targeted skill-building, such as communication, negotiation, and leadership skills, which remain underdeveloped in many WLPs despite their importance for senior-level readiness (55, 67). A further set of recommendations focused on strengthening relational and community-building aspects of New Era. For instance, the adoption of a mentor–mentee matching approach that considers leadership style, professional interests, sport-sector expertise, and developmental needs was identified as having potential to improve outcomes for alumni. This is a pertinent reflection, as relational fit is a strong predictor of mentoring effectiveness and psychological safety (71). Alumni also emphasised the value of ongoing engagement—both within cohorts and across the broader alumni network—to support long-term peer learning and sustained professional relationships. These suggestions echo social network and community-of-practice research, which demonstrates that leadership development is amplified when participants are embedded in ongoing peer learning communities that provide diverse perspectives (67, 72).

Alumni emphasized the need for clearer articulation of the program's goals and intended outcomes at an organizational level. They questioned whether New Era aims to broaden women's participation in leadership, accelerate progression into senior roles, cultivate a sense of community, or pursue all three simultaneously. This ambiguity reflects a common challenge in WLPs, which often do not incorporate explicit theories of change or clearly defined developmental pathways (18, 73, 74).

Where the organizational recommendation targets internal programmed design, participants also highlighted industry-level conditions that shape future participation and opportunities. At an industry level, the establishment of partnerships and sponsorships was flagged as having potential to enhance equity of access to New Era by offsetting travel costs, particularly for alumni outside the UK or working in smaller organizations. Members of the New Era community also recommended strengthening digital infrastructure, including job hubs and online discussion spaces, to ensure continuity and accessibility across global locations. While there are some pitfalls in online mentoring, as outlined above, digital learning ecosystems have been shown to support sustained leadership development in geographically dispersed contexts (75).

Taken together, these findings and recommendations suggest that WLPs in sport should not be designed as individual development interventions alone. While confidence, communication, and leadership skills remain important, WLPs may be most valuable when they also address the relational and organizational conditions that shape leadership progression. This includes creating access to senior leaders, building durable peer and alumni networks, increasing visibility through industry platforms, clarifying mentoring and sponsorship pathways, and reducing practical barriers to participation for those outside dominant geographic and organizational centers.

New Era's value lies in the intentional design of a women-centered developmental space where these elements are integrated. This interpretation resonates with social women-centered learning initiatives (76) and with women-only sport leadership spaces that emphasize connection and collective support (77). For women and non-binary professionals in sport, leadership development requires more than preparing individuals to navigate existing systems. It requires sustained investment, resource allocation, and infrastructures that make sport leadership systems more accessible, visible, and accountable (78).

### 4.5 Strengths and limitations

A major strength is the incorporation of international perspectives, extending women's leadership development literature beyond the predominantly US- and Australia-centric lens that has traditionally shaped this field (11). A further strength is the inclusion of participants working broadly across five sport leadership sectors. This breadth reflects the diversity of the contemporary sports industry and strengthens the relevance and, potentially, the transferability of the findings across varied leadership settings.

Several limitations should also be acknowledged. First, cohort representation was subject to self-selection bias. Participants included 13 alumni and eight Steering Group members, representing approximately 45% of all New Era alumni and 50% of the Steering Group. As with most interview-based research, participation is more likely among individuals willing to discuss potentially personal or professionally sensitive experiences and who may be particularly invested in the topic. It is therefore possible that those who participated had either more positive or more negative experiences of the programmed than those who did not. Although the inclusion of Steering Group members provided a broader perspective on alumni experiences, given that they mentored 2–3 alumni each year, this does not remove the possibility that less engaged or more neutral perspectives were underrepresented. Second, the study relied on participants’ self-reported accounts. The findings should be interpreted as reflecting how participants perceived and described their experiences, rather than objectively measured programmed impacts. For example, although participants reported benefits in career progression, confidence, leadership practice, and influence within their organizations, the study did not assess objective indicators such as promotions, role expansions, retention, policy changes, or measurable improvements in gender equity. Participants’ accounts may have been shaped by recall bias and social desirability bias, particularly where they retained an ongoing affiliation with or sense of commitment to the programmed. In addition, the cross-sectional nature of the study limits the ability to assess how perceived outcomes developed over time or whether they were sustained. The study cannot establish causal relationships between programmed participation and subsequent professional or organizational outcomes (79).

### 4.6 Future research

Considering these limitations, future research on women's leadership programmed should move beyond cross-sectional, retrospective, self-report designs and adopt more longitudinal and multi-level approaches to evaluation. Instead, emerging literature suggests the value of assessing outcomes over time, including career progression, retention, network expansion, changes in leadership responsibilities, organizational practice, and broader sector-level gender-equity indicators. Jowett et al. (10) highlights the value of structured evaluation frameworks in women coaches’ leadership development programmed, particularly in tracking outcomes beyond participants’ initial reactions. Similarly, Stephenson et al. (19) propose a comprehensive framework for evaluating the impact of women's leadership programmed across micro, meso, and macro levels, urging researchers to examine not only short-term changes in confidence or capability but also longer-term institutional and sector impacts. Incorporating multi-level evaluation methods would strengthen the evidence base, offer deeper insights into programmed effectiveness, and enhance the programmer’s capacity to influence the global sports leadership landscape.

Future studies would benefit from collecting data at multiple time points, for example, before, during, and after programme participation, to examine how outcomes develop and whether they are sustained. There would also be value in combining qualitative accounts with more objective indicators, such as promotion, retention, role expansion, organisational policy change, or other measurable markers of leadership progression and gender equity. Attention should also be given to how programme experiences vary across career stages, sport sectors, national contexts, and intersectional identities. Such approaches would strengthen the evidence base and provide a more robust understanding of how women's leadership programmes influence individual careers, organizational cultures, and the wider sport leadership landscape.

## 5 Conclusion

This qualitative evaluation of the New Era programmed provides nuanced insights into how contemporary women's leadership initiatives in sport can support individual development whilst engaging with broader questions of gender equity and systemic change. Drawing on the perspectives of alumni and Steering Group members, findings demonstrate that New Era is perceived as a programmed that meaningfully supports women at critical junctures in their leadership journeys and careers. Through the integration of mentoring, sponsorship-like advocacy, visibility pathways, and community-building, New Era extends beyond traditional “fix-the-woman” approaches and illustrates the potential of more relational, contextually embedded models of women's leadership development in sport. These novel findings directly address the limited attention to learning processes and system navigation highlighted in the literature and respond to calls for more integrated, system-aware approaches (11, 20). Further, findings on ongoing challenges for women in sports leadership demonstrate the need for programs such as New Era to continue to progress towards more equitable leadership landscapes within the sports sector.

## Data Availability

The original contributions presented in the study are included in the article/Supplementary Material, further inquiries can be directed to the corresponding author.
