# Supplementary material for: A qualitative programme evaluation of New Era, a mentorship programme for women leaders in sport
Source: Front Sports Act Living. 2026 Jun 17;8:1813783. doi: 10.3389/fspor.2026.1813783 (PMC13318938; doi:10.3389/fspor.2026.1813783)
Supplement: Supplementary file 1 [file Supplementaryfile1.docx]

**Mentors’ program evaluation**

**Kirkpatrick’s (1998) Four-Level Model-Based Interview Schedule**

(questions adapted from Jowett et al., 2024)

**Reactions** (Impressions of the Program)

- **Main Question:** What are your general impressions of the New Era program from a mentor’s perspective?
- **Prompts:**
- What attracted you, or continues to maintain your engagement with, NEW ERA?
- Which aspects of the program do you think were most effective or least effective for supporting mentees?
- Are there areas of the program you feel could have better supported mentors or mentees?
- How well do you think the program met its objectives in fostering leadership development among mentees?

**Learning** (Knowledge and Skills Gained by Mentees)

- **Main Question:** Based on your interactions, what changes or growth have you observed in your mentee(s) throughout the New Era program?
- **Prompts:**
- Have you noticed specific leadership skills or knowledge that your mentee(s) have developed?
- Can you share an example where a mentee applied a concept or skill from the program?
- How has your mentee(s)' understanding of their role as a leader evolved, in your view?

**Improvement & Application** (Applying Knowledge and Skills by Mentees)

- **Main Question:** How do you perceive your mentee(s) have applied the knowledge and skills gained from the program in their leadership roles or career ambitions?
- **Prompts:**
- In your opinion, how well has the program prepared mentee(s) for new challenges or opportunities in sports leadership?
- What impact do you think the program will have on the future career trajectories of your mentee(s)?

**Results** (Impact on Environment/Organization)

- **Main Question:** From your perspective, what impact has the New Era program had on the broader sporting environment or organizations?
- **Prompts:**
- What support or resources would you recommend to help mentees continue to grow after completing the program?
- Do you believe the program has equipped mentees to drive meaningful change in the sports industry or their organizations?
- How has the program contributed to advancing equity in sports leadership, based on your observations?

**References**

Jowett, S., Slade, K., Gosai, J., & Davis, L. (2024). Women coaches leadership development programme: an evaluation study of programme effectiveness. *Frontiers in Sports and Active Living*, *6*. <https://doi.org/10.3389/fspor.2024.1433787>

Kirkpatrick, D. L. (1998). The Four Levels of Evaluation. In (pp. 95-112). Springer Netherlands. <https://doi.org/10.1007/978-94-011-4850-4_5>
