# Supplementary material for: A qualitative programme evaluation of New Era, a mentorship programme for women leaders in sport
Source: Front Sports Act Living. 2026 Jun 17;8:1813783. doi: 10.3389/fspor.2026.1813783 (PMC13318938; doi:10.3389/fspor.2026.1813783)
Supplement: Supplementary file 2 [file Supplementaryfile2.docx]

**Participants’ program evaluation**

**Kirkpatrick’s (1998) Four-Level Model-Based Interview Schedule**

(questions adapted from Jowett et al., 2024)

**Reactions** (Impressions of the Program)

- - **Main Question**: *What were your general impressions of the New Era program?*
  - **Prompts**:
    - What aspects of the program did you particularly like or dislike?
    - Is there anything you feel could have been done differently, or areas you feel could have received more focus?
    - How well did the program meet your expectations for leadership development?

**Learning** (Knowledge and Skills Acquired)

- - **Main Question**: *What have you learned throughout the New Era program, and what had the greatest impact on you?*
  - **Prompts**:
    - Are there specific leadership skills or knowledge areas you feel you’ve developed?
    - Can you share an example of a concept or insight that stood out for you?
    - How has the program influenced your understanding of your role as a leader?

**Improvement & Application** (Applying Knowledge and Skills)

- - **Main Question**: *How do you see the knowledge and insights gained from the New Era program contributing to your personal ambitions or career goals?*
  - **Prompts**:
    - Have you been able to apply any of the skills or knowledge in your current role or other areas?
    - How has the program prepared you to take on new challenges or opportunities in sports leadership?
    - What impact do you anticipate the program will have on your future career trajectory?

**Results (Impact on Environment/Organization)**

- - **Main Question**: *What recommendations do you have for improving the quality of the New Era program and supporting participants beyond the program?*
  - **Prompts**:
    - What support or resources do you think would help participants continue to grow after the program?
    - Do you believe the program has the potential to drive change in your organization or the broader sports industry?
    - How has the program influenced your ability to contribute to a more equitable environment in sports leadership?

**References**

Jowett, S., Slade, K., Gosai, J., & Davis, L. (2024). Women coaches leadership development programme: an evaluation study of programme effectiveness. *Frontiers in Sports and Active Living*, *6*. <https://doi.org/10.3389/fspor.2024.1433787>

Kirkpatrick, D. L. (1998). The Four Levels of Evaluation. In (pp. 95-112). Springer Netherlands. <https://doi.org/10.1007/978-94-011-4850-4_5>
